# Yb:MoO_3_/Ag/MoO_3_ Multilayer Transparent Top Cathode for Top-Emitting Green Quantum Dot Light-Emitting Diodes

**DOI:** 10.3390/nano10040663

**Published:** 2020-04-02

**Authors:** Chun-Yu Lee, Yi-Min Chen, Yao-Zong Deng, Ya-Pei Kuo, Peng-Yu Chen, Leo Tsai, Ming-Yi Lin

**Affiliations:** 1 AU Optronics Corporation, Hsinchu 30078, Taiwan; d92941010@ntu.edu.tw (C.-Y.L.); joanne.kuo@auo.com (Y.-P.K.); kevinplus2012@gmail.com (P.-Y.C.); zxcvb9012@gmail.com (L.T.); 2 Department of Electrical Engineering, National United University, Miaoli 36003, Taiwan; M0821023@nuu.edu.tw (Y.-M.C.); M0721018@nuu.edu.tw (Y.-Z.D.)

**Keywords:** QLEDs, top-emission, DMD, transparent cathode

## Abstract

In this study, we report on the application of a dielectric/ultra-thin metal/dielectric (DMD) multilayer consisting of ytterbium (Yb)-doped molybdenum oxide (MoO_3_)/silver (Ag)/MoO_3_ stacked as the transparent cathode in top-emitting green quantum dot light-emitting diodes (QLED). By optimizing the Yb doping ratio, we have highly improved the electron injection ability from 0.01 to 0.35. In addition, the dielectric/ultra-thin metal/dielectric (DMD) cathode also shows a low sheet resistance of only 12.2 Ω/sq, which is superior to the resistance of the commercially-available indium tin oxide (ITO) electrode (~15 Ω/sq). The DMD multilayer exhibits a maximum transmittance of 75% and an average transmittance of 70% over the visible range of 400–700 nm. The optimized DMD-based G-QLED has a smaller current leakage at low driving voltage. The optimized DMD-based G-QLED enhances the current density than that of G-QLED with indium zinc oxide (IZO) as a cathode. The fabricated DMD-based G-QLED shows a low turn-on voltage of 2.2 V, a high current efficiency of 38 cd/A, and external quantum efficiency of 9.8. These findings support the fabricated DMD multilayer as a promising cathode for transparent top-emitting diodes.

## 1. Introduction

Recently, quantum dot light-emitting diodes (QLEDs) have gained significant attention as the highest potential candidates for future display applications due to several advantages including size control, emission wavelength tunability, high color purity, narrow linewidth, high photoluminescence quantum yield (PLQY), and solution-processed fabrication [1,2,3,4,5,6]. Since the first report of QLEDs in 1994 [7], many approaches have been studied to fabricate high performance QLEDs, including interfacial engineering, material synthesis, and device architecture [8,9,10,11]. Especially, the top-emitting QLED is an important device structure for high pixels per inch (PPI) display technology, due to its compatibility with fabrication on thin film transistors (TFTs) directly, without sacrificing the aperture ratio. Hence, the top electrodes with high conductivity and transparency in the visible light region are highly desired. Generally, thin metal (Al, Ag, or Au) [12,13,14], metal alloy (Mg:Ag) [15,16,17], and conductive oxides (indium tin oxide (ITO), indium zinc oxide (IZO), and fluorine-doped tin oxide (FTO)) are most frequently used as a transparent electrode [18,19,20]. However, the relatively large absorption, reflection, and surface plasma loss of typical thin metals and metal alloys limit the transparency [21]. As for conductive oxides, their deposition process requires the sputtering method or electron/ion beam-assisted techniques, causing plasma damage of the underlying films [22,23]. This sputtering damage may be alleviated to some degree by using an inserting layer, but complete protection is still a challenge [24,25,26]. Moreover, obtaining high quality oxide film with high transparency and conductivity often requires a high temperature that is harmful to the devices [23,27]. Therefore, several alternative approaches have been explored and proposed by many scientific groups to develop a top electrode [28,29]. 

The use of multilayer electrodes, especially so-called dielectric/ultra-thin metal/dielectric (DMD), one of the most promising structures of the top electrode, not only has high transmittance and low resistance but also can be compatible with thermal evaporation without plasma bombardment. Numerous DMD configurations have been studied to improve its transmittance and conductivity in hopes of competing with conductive oxides [30,31,32]. The outer MoO_3_ significantly reduces the reflectance of the Ag layer. Consequently, the molybdenum (MoO_3_)/Ag/MoO_3_ electrode has shown promise. However, the dielectric materials used in the DMD electrode usually possess p-type characteristics and high work function. This has hindered the progress of applying DMD in top-emitting devices. 

In this paper, we propose to use ytterbium (Yb)-doped MoO_3_/Ag/MoO_3_ as a top cathode to fabricate top-emitting weak-cavity green QLEDs. The Yb-doped inner MoO_3_ is introduced as an efficient electron injection layer (EIL). The Yb:MoO_3_/Ag/MoO_3_ cathode exhibits an average transmittance of above 70% in the visible light region and low sheet resistance around 12.2 Ω/sq. The optical and electrical properties of various Yb doping ratios of the DMD cathode is investigated. Its relationship with luminous efficiency of the G-QLEDs is also studied. Our green QLED with an Yb:MoO_3_/Ag/MoO_3_ top cathode exhibits a current efficiency of 38 cd/A and an external quantum efficiency (EQE) of 9.8% with a color co-ordinate (0.199, 0.742).

## 2. Materials and Methods 

### 2.1. Fabrication of the Dielectric/Ultra-Thin Metal/Dielectric (DMD) Cathode

Prior to beginning the fabrication process, the glass substrates were cleaned in an ultrasonic cleaner successively with deionized water, acetone, and isopropyl alcohol for 20 min in each round. Following the cleaning process, they were dried in a vacuum oven (DLAB Inc., New Taipei, Taiwan) at 100 °C for 10 min. A DMD multilayer cathode of three layers (Yb:MoO_3_/Ag/MoO_3_) was fabricated on a glass substrate using a thermal evaporation system at a vacuum pressure of 3 × 10^−6^ torr, and the thicknesses of the films were monitored during deposition. The inner dielectric layer was prepared by thermal coevaporation of Yb and MoO_3_ with a ratio of 0:1, 0.2:1, 0.6:1, and 1:1 by volume, respectively. Yb is a low work function (WF) metal (WF: 2.63 eV). It is frequently used as a dopant to improve the electron injection. The deposition rates of the Yb were varied from 0.01 to 0.1 nm/s for doping with MoO_3_. The deposition rates of MoO_3_ and Ag were fixed at 0.1 nm/s. The thickness of the DMD multilayer cathode was as follows: Yb:MoO_3_ (5 nm)/Ag (10 nm)/MoO_3_ (32 nm). 

### 2.2. Fabrication of Top-Emitting Green QLEDs

First, we cleaned the ITO/Ag/ITO substrate according to the method mentioned above. All the QLED devices were fabricated in the nitrogen-filled glove box. Then the substrate was treated under UV ozone for 15 min to increase the work function and to improve the adhesion to hole injection layer (HIL). Subsequently, HIL was spin-coated at 910 rpm on the ITO/Ag/ITO substrate and baked at 230 °C for 15 min. HTL was spin-coated at 3000 rpm and baked at 120 °C for 20 min. After that, green quantum dots (QDs) and Zn_0.85_Mg_0.15_O nanoparticles (NPs) were deposited layer-by-layer via spin casting on the HTL/HIL/substrate. Both the green QDs and Zn_0.85_Mg_0.15_O NPs layers were spin casted at 3500 rpm. Finally, the transparent top cathode was deposited by thermal evaporation. All devices were encapsulated in a glass-to-glass epoxy sealed package with desiccant. The emitting area was 2 mm × 2 mm. The QLED devices had the following structures: ITO/Ag/ITO glass (top ITO: 12 nm thick; Ag layer was to reflect light to top electrode), HIL (20 nm thick), HTL (30 nm thick), green QDs (13 nm thick), electron transport layer (ETL) (40 nm thick), and top cathode. HIL is composed of PFSA (tetrafluoroethylene-perfluoro-3,6-dioxa-4-methyl-7-octene-sulfonic acid copolymer), PEDOT:PSS (Poly(3,4-ethylenedioxythiophene)-poly(styrenesulfonate)) and dimethyl sulfoxide. HTL is poly[(9,9-dioctylfluorenyl-2,7-diyl)-co-(4,4′-(N-(4-s-butylphenyl))diphenylamine)] (TFB) derivative. The light emitting layer (EML) and ETL were green QD and Zn_0.85_Mg_0.15_O nanoparticles (NPs), which were purchased from Mesolight Inc. (Suzhou, China) The PLQY of QDs was 80%. Here, Zn_0.85_Mg_0.15_O was composed of Mg-doped ZnO NPs for efficient electron transport and easy injection in the green QD EML layer. 

### 2.3. Fabrication of Electron-Only Devices (EODs)

To investigate the electron injection ability of the DMD cathode, we fabricated the electron-only devices (EODs) by using the same method as our green QLEDs. All the EODs were fabricated in the vacuum chamber. The EODs consisted of ITO/Al/2,9-Dimethyl-4,7-diphenyl-1,10-phenanthroline (BCP)/DMD cathode with different Yb doping ratios of 0, 0.2, 0.6, and 1, respectively, in the inner MoO_3_ layer. The BCP is an efficient electron transporting material with high electron mobility of ~10^−3^ cm^2^/Vs [33], lowest unoccupied molecular orbital (LUMO) level of 3.5 eV, and a very deep highest occupied molecular orbital (HOMO) level of 7.0 eV. The electron transporting property was similar to Zn_0.85_Mg_0.15_O. Finally, the Al and BCP were deposited by thermal evaporation with different masks to define the deposited area. The thicknesses of Al and BCP were 150 and 200 nm, respectively.

## 3. Results and Discussion

**Figure 1:**
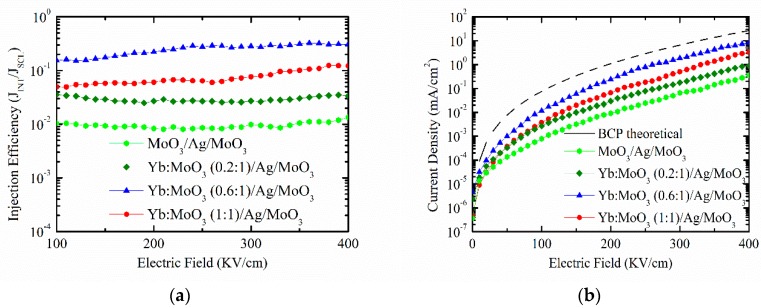
(**a**) Current density electric field characteristics, and (**b**) electron injection efficiency electric field characteristics of the electron-only devices with different Yb doping ratios in inner MoO_3_.

The electron injection ability of the DMD cathode could be quantitatively characterized by analyzing the space charge-limited current of EODs. Figure 1a shows the current density voltage (*J*–*V*) characteristic of EODs with configuration of ITO/Al (150 nm)/BCP (200 nm)/Yb:MoO_3_ (5 nm)/Ag (9 nm)/MoO_3_ (32 nm). The inner MoO_3_ layer was doped with different Yb ratios of 0.2, 0.6, and 1, respectively. The undoped inner MoO_3_ layer was also included for comparison. The theoretical space charge-limited current density (*J*_SCLC_) was calculated from the following equation (Equation (1)) [34],

(1) $$J=\frac{9}{8}\varepsilon \varepsilon_{o}\mu \frac{E^{2}}{L}$$

where *ε* was relative permittivity (assumed to be 3.0), *ε*_0_ was vacuum permittivity (8.854 × 10^−14^ F/cm), *μ* was mobility of the current carrier, *E* was the applied electric field across the sample, and *L* was the thickness of the BCP (200 nm). Charge carrier mobility showed field dependency, which was understood in terms of hopping transport. Field dependency was generally expressed by the Poole–Frenkel equation (Equation (2)),

(2) $$\mu =\mu_{o}\exp\left(\gamma \sqrt{E}\right)$$

where *μ*_0_ was the zero-field mobility and *γ* was the Poole–Frenkel factor. By combining Equations (1) and (2), we could express the field-dependent space charge-limited current density (SCLC) as Equation (3).

(3) $$J=\frac{9}{8}\varepsilon \varepsilon_{o}\frac{E^{2}}{L}\mu_{o}\exp\left(0.89\gamma \sqrt{E}\right)$$

The Poole–Frenkel factor *γ* was the field-dependent mobility coefficient of BCP (in this case). *γ*, obtained from literature [33], was found to be 0.011. Finally, the theoretical steady-state *J*–*V* characteristic for BCP is depicted in Figure 1a (black dashed line).

As shown in Figure 1a, the current density was enhanced significantly as the Yb doping ratio increased from 0.2 to 0.6. It is clear that the current density of EOD reached the highest and closest value to the current of theoretical value of BCP. As for the inner MoO_3_ without Yb doping, the EOD showed the lowest current density. It is anticipated that Yb doping into MoO_3_ leads to lower work function and enhancement of the electron injection. Hence, we can say that the enhanced capability of the current density of the EODs was mainly attributed to the improved injection ability of Yb-doped MoO_3_. However, when the Yb doping ratio was further increased to 1, the current density was degraded. We can consider that the morphology of the Yb:MoO_3_ layer (1:1) deteriorated.

The electron injection efficiency (*η*_INJ_) could be obtained from Equation (4),

(4) $$\eta_{\mathrm{INJ}}=J_{\mathrm{INJ}}/J_{\mathrm{SCLC}}$$

where *J*_INJ_ was the measured steady-state current density and *J*_SCLC_ was theoretical space charge-limited current density. Figure 1b displays the electron injection efficiency (*η*_INJ_) curves of the EODs with different Yb doping ratios in inner MoO_3_. The electron injection efficiency (*η*_INJ_) of the EOD with Yb:MoO_3_ (0.6:1) as an inner layer was about 0.35, while the maximum injection efficiency of the EOD with undoped MoO_3_ was at the order of 10^−2^. This shows that the Yb:MoO_3_ doping ratio (0.6:1) had a superior electron injection property of the DMD cathode. In other words, this DMD electrode can be used as a high performing cathode in top-emitting diodes.

**Figure 2:**
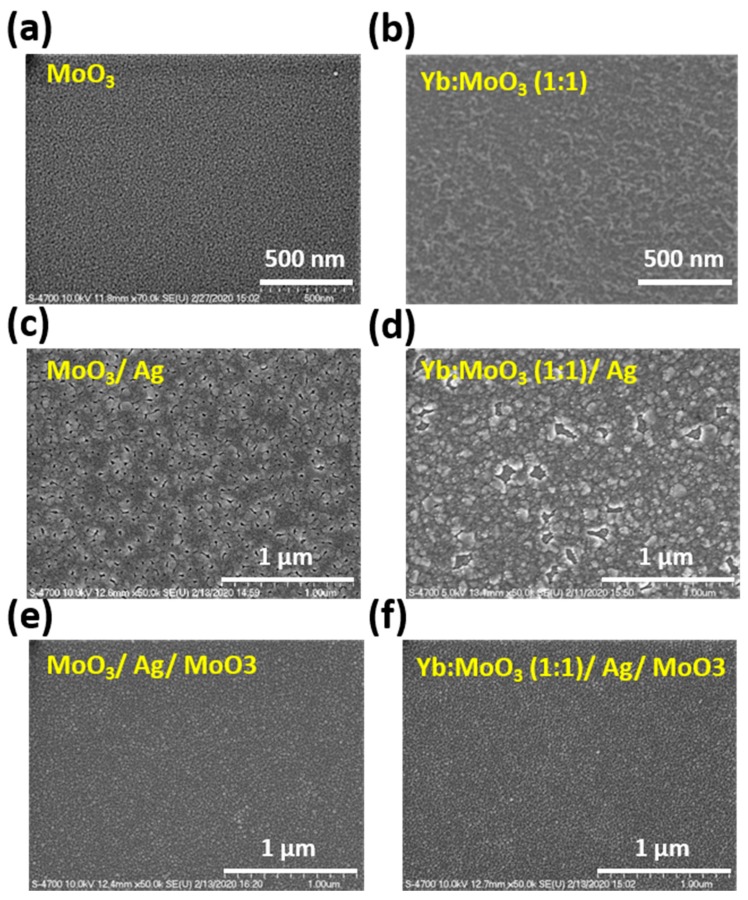
Scanning electron microscopy (SEM) images of the DMD surface morphology on glass substrate. (**a**) MoO_3_ (5 nm), (**b**) MoO_3_ (5 nm)/Ag (10 nm), (**c**) MoO_3_ (5 nm)/Ag (10 nm)/MoO_3_ (32 nm), (**d**) Yb:MoO_3_ (1:1–5 nm), (**e**) Yb:MoO_3_ (1:1–5 nm)/Ag (10 nm), (**f**) Yb:MoO_3_ (1:1–5 nm)/Ag (10 nm)/MoO_3_ (32 nm).

To further verify that the degraded electron injection current was related to the deteriorated morphology of Yb:MoO_3_ by increasing the Yb doping ratio excessively, we examined the surface morphologies of the sequentially-deposited DMD multilayers with and without Yb doping in inner MoO_3_. The corresponding scanning electron microscopy (SEM) (Hitachi, Ltd., Tokyo, Japan) images of the DMD multilayers are shown in Figure 2. Initially, MoO_3_ (5 nm) exhibited an absolutely smooth morphology (Figure 2a). Subsequently, the morphology was remarkably changed to void-area coverage after 5 nm of Ag film was deposited on MoO_3_ (Figure 2b). The void size was about 20–100 nm. This was because Ag film tends to accumulate and form cone-like agglomerates at such a thickness. When capping a 32 nm of MoO_3_, the surface coverage of the DMD multilayers was obviously improved (Figure 2c). In contrast, Yb:MoO_3_ (1:1, 5 nm) showed a very rough surface (Figure 2d). This indicates that Yb doping into MoO_3_ degraded the film uniformity. The poor uniformity led to the addition of scattering centers which reduced the injection current. When 5 nm of Ag film was deposited on Yb:MoO_3_, the void-area coverage significantly deteriorated (Figure 2e). The void size was raised to about 100–200 nm. It is understood that the surface roughness of the Yb:MoO_3_ underlayer had a great effect on the uniformity of the following Ag layer, further influencing the DMD characteristics. Finally, the morphology of the DMD multilayers with Yb-doped inner MoO_3_ was effectively improved after capping the top MoO_3_ (Figure 2f). Compared with the DMD with undoped inner MoO_3_, the morphology was almost the same. However, we can consider that the morphology of the inner MoO_3_ was critical to influencing the device performance due to direct contact with ETL.

**Figure 3:**
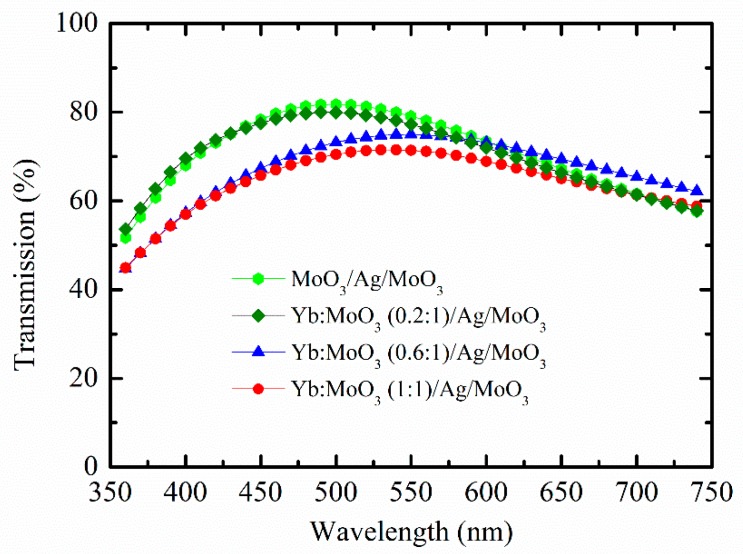
Measured optical transmittance of the DMD multilayers on glass substrate.

The characterization of transmittance and sheet resistance of the DMD multilayer with different Yb doping ratios was explored. Figure 3 shows the variations of transmittance as a function of wavelength in the range of 350–750 nm. The shape of the transmittance spectrum could be determined by the Yb doping ratio. Transmittance decreased as Yb doping ratio increased. This indicates that Yb doping into MoO_3_ led to scattering losses because of the uniformity deterioration of the morphology. The results were consistent with the morphology of the DMD multilayer investigation. When Yb doping ratio was raised from 0 to 0.6, transmittance decreased quickly in the wavelength range of 350–525 nm. Similarly, when Yb doping ratio was raised from 0.2 to 1, transmittance decreased rapidly in the wavelength range of 525–750 nm. The DMD multilayer with undoped inner MoO_3_ had the highest average transmittance (400–700 nm) of 74.1% while the DMD multilayer with Yb:MoO_3_ (1:1) had the lowest average transmittance of 66.7%. With an appropriate Yb doping ratio into MoO_3_ (0.6:1), transmittance could be maintained above 70% for top-emitting diodes.

For the electric measurement of the DMD multilayer, the sheet resistance monotonically increased from 8.3 to 13.1 Ω/sq with the increase of Yb doping ratio from 0 to 1. Compared with conventional commercially-available ITO (~15 Ω/sq), the DMD multilayer provided superior conductivity. As a comparison, the measured transmittance and sheet resistance of the DMD multilayer are summarized in Table 1. Based on these results (Figure 1 and Table 1), we considered Yb:MoO_3_ (0.6:1) to be suitable for our DMD cathode because of its outstanding electron injection ability, transparency, and electrical resistance.

**Figure 4:**
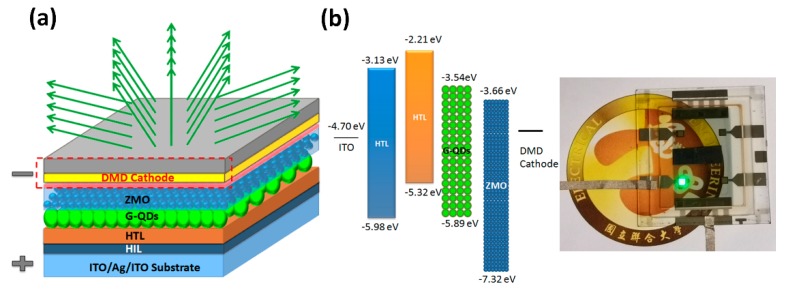
(**a**) Schematic of the device structure. (**b**) Energy band diagram of the red top-emission QLEDs. Photograph of the working device.

In this report, our top-emitting green QLED devices consisted of a patterned ITO/Ag/ITO glass, HIL (20 nm), HTL (30 nm), QD EML (13 nm), ETL (40 nm), and a DMD cathode (5 nm/10 nm/32 nm), as schematically shown in Figure 4a. Figure 1b shows the schematic diagram of a fabricated green top-emission QLED structure with an energy band/level diagram of each layer. The electronic energy levels were investigated by ultraviolet photoemission spectroscopy (UPS) in a Kratos AXIS Ultra-DLD ultrahigh vacuum photoemission spectroscopy system with HeI excitation. The photo in Figure 4b shows our top-emitting green QLED with a DMD cathode Yb:MoO3 (0.6:1) demonstrating the emission quality of the transparent top cathode.

**Figure 5:**
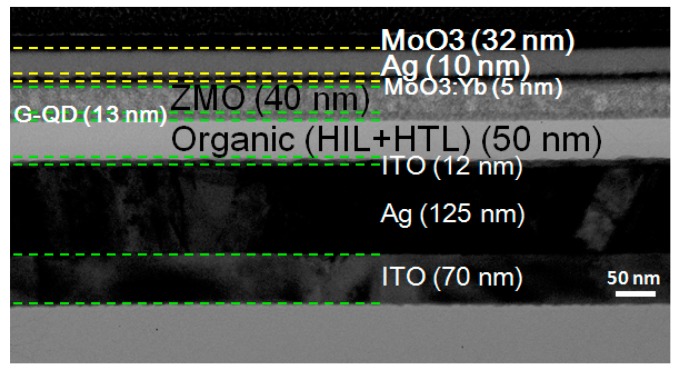
Cross-sectional transmission electron microscopy image of our green top-emission QLED with a DMD cathode.

Figure 5 shows the corresponding cross-sectional transmission electron microscopy (TEM) image of our green top-emission QLED with a DMD cathode. The figure clearly shows that a compact QD-emitting layer was placed between the charge carrier transport layers and a DMD cathode was deposited on the ETL (Zn_0.85_Mg_0.15_O).

**Figure 6:**
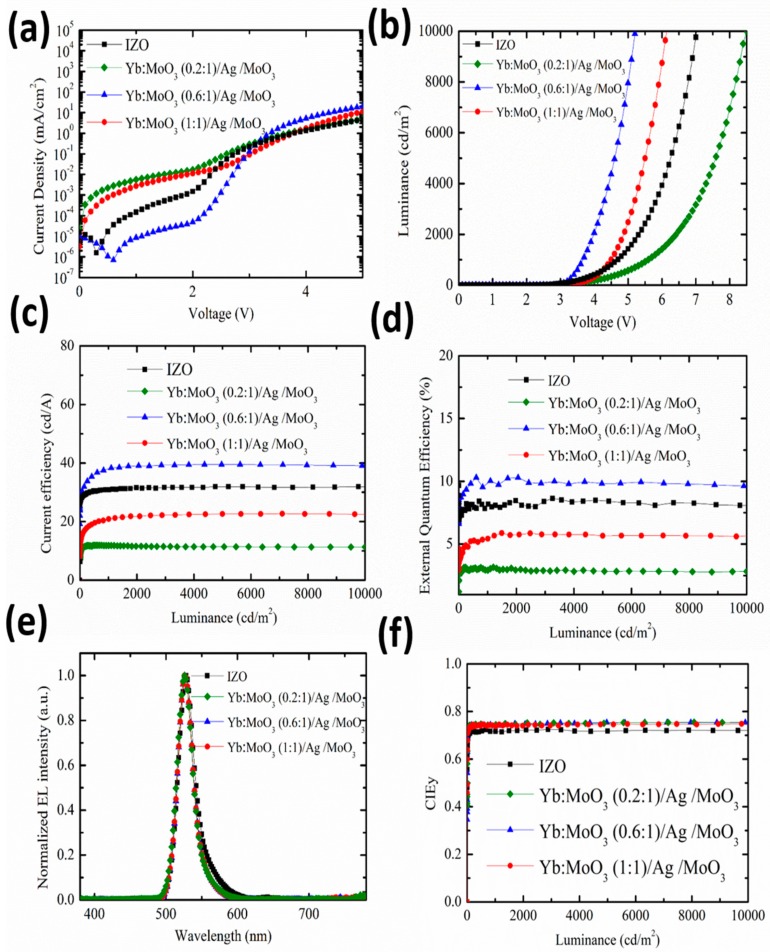
Device performance of the green top-emission QLEDs with different top transparent cathodes. (**a**) Current density voltage (*J*–*V*) characteristics. (**b**) Luminance current density (*L*–*J*) characteristics. (**c**) Current efficiency luminance characteristics. (**d**) EQE luminance characteristics. (**e**) Normalized EL spectra. (**f**) CIEy luminance characteristics.

In this study, to further investigate the effect of the DMD cathode, QLEDs with different Yb doping ratios in inner MoO_3_ for the DMD multilayer were fabricated. The device with IZO as a cathode was also fabricated for comparison, and all the other layers had identical thickness to the DMD-based device. Figure 6 shows the EL performance of the fabricated QLEDs. *J*–*V* characteristics are shown in Figure 6a; the current density was raised with increasing Yb doping ratio from 0.2 to 0.6. It is clear that the device with a Yb doping ratio of 0.2 showed very serious current leakage at low driving voltage (<2.5 V). This was attributed to the high electron injection barrier between MoO_3_ and ETL due the inherent p-type property of MoO_3_. Consequently, when the Yb doping ratio was increased to 0.6, the device showed the highest current density at the same voltage. The device exhibited very good diode characteristics without leakage at low driving voltage. In addition, when driving voltage was increased (>3 V), the slope of the current density increased with the raising of the Yb doping ratio. This can be interpreted as a result of work function-modifying by Yb doping into MoO_3_. The Yb doping into MoO_3_ led to the reduction of the electron injection barrier. This phenomenon resembles the work reported in the literature [34]. For the device with a Yb doping ratio of 1, the degradation of current density was obviously higher than that of the device with a Yb doping ratio of 0.6. Moreover, the device also showed the high current leakage at low driving voltage. We attributed this phenomenon to the uniformity deterioration of the Yb:MoO_3_ morphology of the DMD cathode. In other words, even though the devices with Yb doping possessed a high slope of current density, which helped electron injection, an excessive doping ratio degraded the current density. The above results are consistent with the measurements of EOD characteristics in Figure 1. Compared with the device with the IZO cathode, the device with the DMD cathode with an appropriate Yb doping ratio (0.6) into MoO_3_ showed more superior and stable *J*–*V* characteristics. This can be attributed to the thermal evaporation process, and it was suitable to be used as a top transparent cathode without plasma damage. In other words, the DMD cathode with appropriate Yb doping ratio (0.6) into MoO_3_ was a more appropriate electrode for top-emitting diodes compared with an IZO cathode (160 nm). Figure 6b–d demonstrates the luminescence current density (*L*–*J*) characteristics and current efficiency luminescence EQE properties of these devices. As expected, the highest device performance was observed with the device that had a Yb doping ratio of 0.6, while the lowest device performance was shown with the device that had undoped DMD (not shown here). It should be noted that the DMD with undoped inner MoO_3_ possessed p-type characteristics, leading to the device with large turn-on voltage and unstable EL characteristics. In addition, it is worth noting that the device with the DMD cathode with an appropriate Yb doping ratio (0.6) into MoO_3_ showed a 22.9% enhanced current efficiency from 30.9 to 38 cd/A compared to the IZO cathode counterparts. The corresponding EQE also showed 21% enhancement from 8.1% to 9.8%. It should be noted that the optical structure of the device was not optimized, and could be further adjusted to obtain better device performance for our DMD G-QLEDs. Figure 6e illustrates the electroluminescence spectra of QLEDs. All the DMD-based devices exhibited the same peak wavelength (528 nm) and full-width at half-maximum (FWHM), and these devices showed similar Commission Internationale de L’Eclairage (CIE) properties (see Figure 6f). The difference between DMD-based and IZO-based devices in the EL spectrum and CIEy resulted from the difference in the optical cavity. Owing to CIE 1931 color co-ordinates of (0.199, 0.742) and a narrow FWHM of 25 nm, the color saturation makes this green top-emission QLED an ideal green array for display application. A summary of the detailed EL performance of all green top-emission QLED devices is listed in Table 2.

## 4. Conclusions

We utilized a Yb-doped MoO_3_ inner layer to enhance the electron injection efficiency of a DMD electrode for top-emitting green QLEDs. The ratio of Yb:doping in the inner MoO_3_ was optimized based on the electron injection ability, film morphology, and transmittance. We were able to confirm the optimized ratio (0.6:1). It is shown that the Yb doping ratio can affect the surface morphology of Yb:MoO_3_ and Yb:MoO_3_/Ag, leading to a change in the electron injection ability and transmittance. The fabricated DMD cathode showed potential not only for its electron injection ability but also for its average transmittance above 70% in the wavelength of 400–700 nm. By using an appropriate Yb doping ratio, we achieved a high current efficiency of 38 cd/A with an external quantum efficiency of 9.8% in our green top-emission QLEDs. Compared to the device with IZO as a top cathode, this showed a 22.9% enhancement of the current efficiency.

## Figures and Tables

**Table 1:** Sheet resistance and optical transmittance with different Yb doping ratios in inner MoO_3_.

	Sheet Resistance (Ω/sq) (5 Point Average)	T% (400–700 nm)	T% (Peak)
MoO_3_/Ag/MoO_3_	8.3	74.1	81.7
Yb:MoO_3_ (0.2:1)/Ag/MoO_3_	8.4	73.1	80.0
Yb:MoO_3_ (0.6:1)/Ag/MoO_3_	12.2	70.5	75.0
Yb:MoO_3_ (1:1)/Ag/MoO_3_	13.1	66.7	71.5

**Table 2:** Summaries of 1931 CIE (x, y) chromaticity co-ordinates, electroluminescence emission peak wavelength (*λ*_max_), FWHM, turn-on voltage (*V*_T_), current efficiency (*η*_A_), and external quantum efficiency (*η*_EQE_) of the green top-emission QLEDs with different top transparent cathodes. Turn-on voltage is measured at 1 cd/m^2^.

Top-Emitting G-QLED	x	y	l	FWHM	*V*_T_	cd/A	EQE
	--	--	nm	nm	V [1 cd/m^2^]	1000 nits	
**Yb:MoO_3_ (0.2:1)/Ag/MoO_3_**	0.233	0.718	528	25	2.2	11.8	2.9
**Yb:MoO_3_ (0.6:1)/Ag/MoO_3_**	0.199	0.742	528	25	2.2	38.0	9.8
**Yb:MoO_3_ (1:1)/Ag/MoO_3_**	0.197	0.744	528	25	2.3	28.0	7.4
**IZO**	0.196	0.739	529	26	2.2	30.9	8.1
